# Alcohol, health education and changing notions of risk in Britain, 1980–1990

**DOI:** 10.1080/09687637.2020.1724264

**Published:** 2020-02-11

**Authors:** Alex Mold

**Affiliations:** Centre for History in Public Health, London School of Hygiene and Tropical Medicine, London, UK

**Keywords:** Alcohol, risk, history, health education

## Abstract

This article explores the contentious definition and communication of alcohol consumption limits and their relationship to ideas about risk through an analysis of the development of health education materials during the 1980s. It argues that changing ideas about alcohol and risk, and their communication to the public, were a reflection of both specific developments in thinking about alcohol and the harm it could pose as well as broader shifts within public health policy, practice and outlook. Risk was understood as something experienced by individuals and populations, a conceptual framing that suggested different approaches. To get to grips with these issues, the article focuses on: (1) the definition of alcohol consumption limits; (2) the communication of these limits; and (3) the limits to limits. The problems experienced in defining and communicating limits suggests not only a ‘limit to limits’ but also to the entire notion of risk-based ‘sensible’ drinking as a strategy for health education.

## Introduction

The idea that alcohol poses a danger to health and society is not a new one but from the 1970s onwards the risks associated with drinking began to feature in alcohol health education campaigns in Britain. Mass media advertising, including posters, television advertisements and other material, focused on some of the risks connected with excessive alcohol consumption, such as drunkenness, liver damage, hangovers and the neglect of children. The types of risk emphasised by such campaigns, and who was thought to be at risk from what, changed over time. In the 1970s, health education material was designed primarily to reach alcoholics or heavy drinkers. The language of risk was not always explicit in such campaigns, but where it appeared it tended to focus on health risks to the individual as well as the social and economic consequences of their behaviour. By the late 1980s, campaigns were directed at all drinkers and intended to foster ‘sensible’ drinking within ‘safe’ limits. Risk was something experienced by a much wider population of drinkers.

This article will explore the contentious definition and communication of alcohol consumption limits and their relationship to ideas about risk through an analysis of the development of health education materials during the 1980s. It will argue that changing ideas about alcohol and risk, and their communication to the public, were a reflection of both specific developments in thinking about alcohol and the harm it could pose as well as broader shifts within public health policy, practice and outlook. On the one hand, risk was something that individuals were increasingly expected to manage. Consuming alcohol within ‘safe’ or ‘sensible’ limits was a way to mitigate personal risk. But on the other hand, the risks from alcohol consumption were also understood to exist at the population level. Health education was thought to be of value in reducing alcohol consumption across the population, but other measures, including price controls, and the restriction of alcohol advertising, were also considered. These measures, however, proved to be too contentious, and the strategy of encouraging individuals to consume alcohol within ‘sensible’ limits persisted.

To get to grips with such issues, this article will begin with a discussion of the place of ideas about risk within public health from the 1960s onwards. The application of such ideas contributed towards the framing of alcohol as a public health issue. This was in contrast to earlier decades, when alcohol was primarily a medical or social concern. A series of health education campaigns to address alcohol consumption were mounted during 1970s, and these are briefly examined. The main body of the article focuses on the notion of sensible drinking limits and their relationship to ideas about risk. Drawing on papers in the National Archives which detail the debates between civil servants and the Health Education Council (HEC) over alcohol health education policy, the article will begin by assessing how sensible drinking limits were defined during the 1980s. The article will then go on to look at the ways in which these limits were communicated to the public through an analysis of the production of HEC/Health Education Authority pamphlets on alcohol consumption, as well as an early iteration of the Drinkwise campaign. In the final section, the article will examine some of the critiques of health education for dealing with the risks posed by alcohol and look at some of alternative approaches put forward. The HEC itself recognised that health education alone could not have a significant impact on reducing alcohol consumption. This suggests not only a limit to limits but also to the value of the concept of risk in communicating with the public about how to address alcohol related problems.

## Risk and the framing of alcohol as public health problem

In the post-war period, risk came to occupy a central place in modern society and in the philosophy and practice of public health. A series of epidemiological studies in the USA, the UK, and in other parts of Europe identified specific factors that appeared to increase the likelihood of individuals developing certain kinds of diseases (Rose, 1985). For instance, high cholesterol and high blood pressure were linked to heart disease and cigarette smoking to lung cancer. These characteristics and the behaviours associated with them were described as ‘risk factors’ (Giroux, 2013; Rothstein, 2003). By the 1960s, risk-factor epidemiology was a key part of public health research, and it quickly filtered into public health practice, with multiple efforts made to identify and address risk factors within the population (Weisz, 2014). The concept and application of population-based understandings of risk were further refined by the epidemiologist Geoffrey Rose in the mid-1980s. Rose argued that most ill-health was experienced not by those at high risk, but by those in low risk groups, simply because there were more of them. To prevent disease, the whole population should be targeted, thus reducing the risk for everyone, not just those in high risk groups (Rose, 1992).

Intersecting with this specific sense of epidemiological risk was a broader conceptualisation of risk and its place in modern society. Ulrich Beck, in *The Risk Society*, argued that new technologies posed risks to individuals and societies on an unprecedented scale (Beck, 1992). Anthony Giddens suggested that there were two kinds of risks: ‘external risks’ which could strike individuals unexpectedly from outside but were predictable enough to be insurable. The second kind of risk was ‘manufactured risk’, risks that were created by progress, and especially science and technology. ‘Manufactured risk’ was thus a feature of modern, or rather, late/post-modern societies (Giddens, 1999). Responding to such risks became a key task for government in all of its domains. In public health, this can be seen in the centrality of risk to what has been described as the ‘new public health’. The ‘new public health’ incorporated the findings of risk factor epidemiology and the broader discussions about risk, individual behaviour and the environment to arrive at a specific formulation of the public, its health and what should be done about it (Petersen & Lupton, 1996). This emphasised individual responsibility for dealing with and avoiding health risks (Armstrong, 2009; Ayo, 2012; Crawford, 1980). Health risks were something for the ‘entrepreneurial self’ to manage, especially through behaviour change such as giving up smoking or reducing alcohol consumption (Petersen, 1997).

Ideas about risk at both the individual and population level were central to the reframing of alcohol use as a public health problem. The potential negative consequences of the consumption of alcohol for the health of individuals were well known for centuries, but the conception of alcohol as a danger to public health is a more recent invention. Notions of alcoholism as a disease became widespread in medical circles during the nineteenth century, but this tended to be seen as an individual problem, albeit one with some social consequences (Valverde, 1998). Even as the number of alcoholics appeared to increase during the early 1950s, this was dealt with as an issue requiring individual treatment, rather than as a public health concern (Thom & Berridge, 1995). It was not until the 1960s that a specific sense of the danger alcohol could pose to public health began to develop. This can be observed first in efforts to deal with drink driving. The introduction of the breathalyser in 1967 was intended to protect the public from intoxicated drivers and reduce the number of car accidents (Luckin, 2010a, 2010b). Information campaigns concentrated on the amount of alcohol consumed and the increased risk of road traffic accidents caused by drink-drivers, not about the danger alcohol posed to health per se. A more distinct public health approach to alcohol began to emerge towards the end of the 1960s, as it became clear that there had been a marked rise in alcohol consumption, and with it an increase in alcohol-related illnesses such as cirrhosis of the liver (Nicholls, 2009). The amount of alcohol consumed increased from 5.2 litres of pure alcohol per person in 1950, to 9.3 litres of alcohol per person by the mid-1970s (Royal College of Psychiatrists, 1986, p. 108). Deaths from liver cirrhosis also rose from just over 20 per million in 1950, to more than 40 per million by 1970 (Royal College of Physicians, 1987, p. 24).

**Figure 1:**
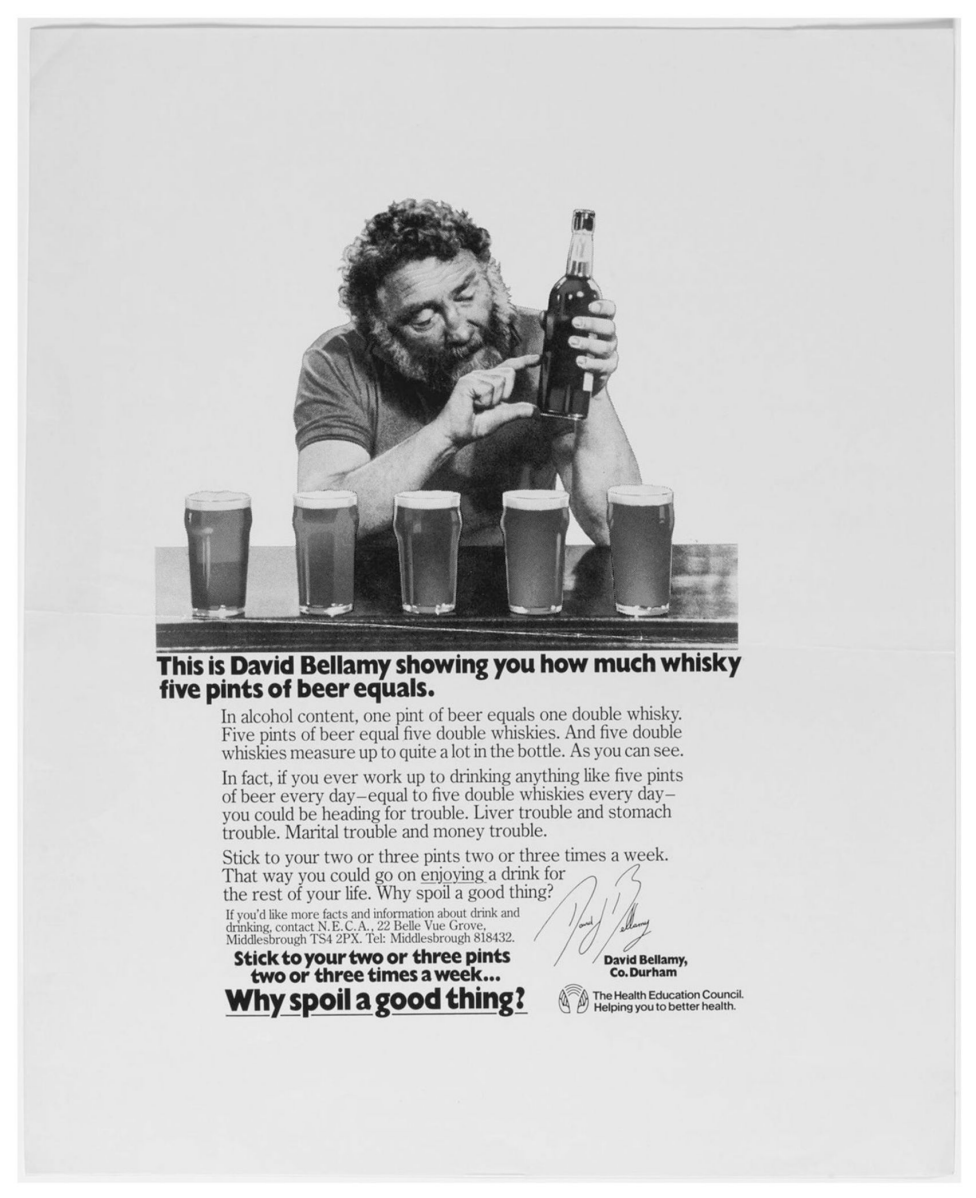
‘Why spoil a good thing?’, Redlands for the Health Education Council, 1981. Image courtesy of the Science Museum Group. This image is released under a Creative Commons Attribution-NonCommercial-ShareAlike 4.0 Licence.

In response to rising concern about the potential risks to health posed by alcohol, in 1973 the HEC decided to pilot a health education campaign in the North East of England (Health Education Council [HEC], 1974). The campaign ran between 1973 and 1981, and consisted of three distinct phases (Mold, 2017). In the first phase, the primary target group were alcoholics. Campaign materials did not contain a specific reference to risk, or indeed any of the health consequences of alcohol consumption beyond drunkenness. There was some information about where to get help, and the suggestion that viewers should ‘drink in moderation’, but there was no indication about what this moderate drinking would look like or why it was necessary. The second phase of the campaign appeared to be aimed at ordinary (albeit ‘heavy’ or ‘excessive’) drinkers rather than alcoholics. Once more, the language of risk was largely absent, although campaign posters did give a very rough sense of what might be considered an excessive amount of alcohol to consume (eight pints of beer and four large whiskies), but the emphasis was on the possible consequences of this for others, not the drinker. In the final phase, the campaign posters, featuring the TV presenter and botanist, David Bellamy, offered guidance on how much alcohol was ‘too much’ (five pints of beer or more) and also suggested a level of moderate consumption as being ‘something like two or three pints two or three times a week.’ Indeed, the benefits of moderate alcohol consumption were tacitly acknowledged by the campaign’s tagline ‘Why spoil a good thing?’ (Figure 1). Some of the potential health risks associated with heavy drinking were acknowledged, such as ‘liver trouble’ and ‘stomach trouble’ as well as the social consequences including ‘marital trouble’ and ‘money trouble.’ Risk thus featured more prominently than in the other phases of the campaign. A further change appeared to revolve around the target group. This campaign was intended to foster moderate drinking amongst all drinkers, and not just alcoholics or heavy drinkers. Such a shift was a reflection of a move within the alcohol policy community to regard alcohol consumption as a population level issue but was also representative of a wider understanding of risk, where this should be located and how it should be managed. How did this play out in subsequent attempts to persuade the public to reduce their consumption of alcohol?

## Defining limits

The promotion of moderate or ‘sensible’ alcohol consumption became a key feature of alcohol policy and health education in the 1980s and 1990s. Devising guidance around what ‘sensible’ levels of alcohol consumption consisted of was, however, problematic. In 1979, the Royal College of Psychiatrists recommended that four pints, four double whiskies or one bottle of wine a day ‘constitute reasonable guidelines of the upper limit of drinking’ (Royal College of Psychiatrists, 1979, p. 139–140). The Department of Health and Social Security’s (DHSS) report, *Drinking Sensibly*, published in 1981, echoed the Royal College of Psychiatrist’s recommendations, but the report also pointed out ‘drawbacks’ to establishing alcohol consumption guidelines. This included the varied effect of alcohol on different people or that fact that people might drink up to suggested limits in the belief that such behaviour was ‘safe’ (Department of Health, 1981, p. 32). Irrespective of the wisdom or otherwise of determining an absolute limit on alcohol consumption, the report advocated an approach which focused on encouraging individuals to manage the risks associated with drinking. Although measures like using taxation to increase the cost of alcohol and other price disincentives were discussed, *Drinking Sensibly* did not endorse such policies. Instead, the report recommended the introduction of more health education efforts orientated around the promotion of ‘sensible drinking’.

The notion of ‘sensible drinking’ was rooted in a wider shift within public health thinking and practice that aimed to foster ‘sensible’ health-related behaviour. In *Drinking Sensibly* the ‘sensible drinker’ was also a ‘responsible citizen’ who must consider ‘what they themselves can do to limit the harm to their own health and the health of others’ (Department of Health, 1981, p. 8). This description of ‘responsible’ behaviour and self risk management was indicative of a particular view of the individual often associated with the ‘new public health’ (Petersen & Lupton, 1996). Members of the public were seen as individuals capable of self-government in response to expert advice (Armstrong, 2009; Crawford, 1980). Such a view placed greater responsibility for dealing with ill-health on the individual, rather than the state or health professionals (Ayo, 2012). Health risks were something for the ‘entrepreneurial self’ to manage, especially through behaviour change such as giving up smoking or reducing alcohol consumption (Petersen, 1997). Encouraging ‘sensible’ behaviour was a way to balance risk but also permit individual choice (Herrick, 2011).

The setting of ‘sensible’ drinking limits and the communication of these to the public fitted within such an approach. Although there had been an attempt to define ‘sensible’ drinking levels by the Royal College of Psychiatrists and in *Drinking Sensibly*, official guidance on this issue first appeared in 1984 when the HEC published a booklet entitled *That*’*s the Limit*. Discussions between the DHSS and the HEC over the production of this guidance during the early 1980s reveals considerable difficulty over determining what the recommended limits should be. There were differences of opinion between the DHSS and the HEC over both the suggested limit and how the risks associated with drinking alcohol should be framed. At first, the DHSS were reluctant to set a nationally agreed safe limit for alcohol consumption at all. Notes from an internal meeting in 1982 stated that ‘There was also uneasiness about the wisdom of suggesting safe levels, and the North East campaign had shown that advice on sensible drinking needed to be related to the patterns of dirnking [sic] and habits considered normal in different regions’ (DHSS, 1982b). The DHSS’s position, as expressed in a meeting with HEC representatives in 1983, was that ‘Alcohol consumption, in moderation, was not itself considered harmful’ (DHSS, 1983g).

The difficulty, of course, was to determine what ‘moderation’ consisted of and on what basis this should be determined. In a draft pamphlet, entitled ‘How much is too much? The facts about alcohol’, the HEC suggested that a ‘safe limit’ consisted of ‘Two or three pints (or their equivalent) two or three times a week. Less for women and less if you’re lighter or shorter than average.’ The draft pamphlet also asserted that ‘You should be especially careful if you are in one of the high risk groups. These include young people, women, the elderly’ (HEC, 1983). The DHSS officials that reviewed the draft were unhappy with both the suggested limit and the HEC’s representation of risk. One civil servant questioned how the HEC had arrived at the limit of two to three pints (or equivalent) two to three times a week, noting only that this was consistent with the message of the Bellamy campaign. He argued that ‘Safe limits are very difficult indeed to recommend and it is worrying that these are not only presented in a rigid uncritical way but also in a manner which does not select limits which have been suggested elsewhere and which seem to exaggerate risks.’ The DHSS were aware that the setting of limits was ‘arbitrary’ and ‘in any case the evidence on which they are based is not yet as good as we would wish’ (DHSS, 1983h). An official also pointed out that ‘it is grossly inaccurate to include women and the elderly among “at risk groups”. What evidence there is suggests these are in reality at a low risk.’ The official concluded that the impression given in the draft guidelines was that the ‘only sensible drinking is no drinking and this is both counter to policy and fact’ (DHSS, 1983e). A second official commented that ‘I think it likely that the drinks industry would ask Ministers if it was departmental policy that safe limits should be prescribed, and on what evidence they were based’ (DHSS, 1983f). In developing advice on sensible drinking levels, the DHSS felt that it was necessary to take into account the wider position of alcohol within society and the interests of other actors, including the alcohol industry, as well as the evidence (or lack thereof) about the risks it posed.

## Communicating limits

The complexities surrounding sensible alcohol consumption levels carried over into the health education materials that were actually produced and distributed to the public. Examining these in some depth points to ongoing issues with defining sensible or safe limits to alcohol consumption, but also how these should be described, what action should be taken and how this related to risk. Following extensive discussions between the HEC and the DHSS, official guidance on alcohol consumption levels appeared in 1984 in the pamphlet *That*’*s the Limit*. The pamphlet recognized that many people enjoyed drinking alcohol and that there was ‘probably’ ‘nothing wrong’ with a drink ‘now and then’. Nonetheless, ‘everybody’ who drank was at ‘risk’ (HEC, 1984). Yet, *That*’*s the Limit* was somewhat vague about what these risks were. The booklet mentioned hangovers and accidents, as well as ‘damage to your health, to your family and to your self-esteem’ but these risks were not spelled out in any detail. Later in the pamphlet, there was an attempt to correlate drinking levels with potential harm. *That’s the Limit* set out ‘safe limits’ for drinking. These were defined as two to three pints two to three times a week for men, and two to three ‘standard drinks’, two to three times a week for women. The pamphlet stated that ‘too much’ alcohol consisted of 56 ‘standard drinks’ a week for men and 35 ‘standard drinks’ for women. Individuals consuming alcohol above this level were told that ‘It is rare for anybody drinking as much as this not to be harming themselves’. This harm included damage to the ‘liver, brain, heart or nervous system’ as well as the potential for dependence and personal problems such as damage to relationships and financial difficulties. The guidelines established by *That*’*s the Limit* represented a more precise sense of what excessive alcohol consumption consisted of than previously, but there was still some ambiguity. It was unclear, for instance, exactly what a ‘standard drink’ consisted of. Readers were told this equated to a single measure of spirits or half a pint of beer, or a ‘small’ glass of sherry or a ‘glass’ of wine. There was no indication of the relative size of the glasses or the strength of alcohol these contained.

Three years after the publication of *That*’*s the Limit* a new version appeared, issued by the HEC’s successor organisation, the Health Education Authority (HEA). This booklet contained similar content, with a few significant changes. The title of the pamphlet remained the same, but the cartoon character on the front cover asked readers ‘What is your limit?’ instead of ‘What is the limit?’ This more personalised message gave a less absolute sense of ‘the limit’ to alcohol consumption and acknowledged that this might vary from person to person. The mode of address also suggested that alcohol consumption was something the individual should take responsibility for. At the same time, the new version of the pamphlet also provided a more specific sense of what an absolute limit might consist of. ‘Standard drinks’ were replaced by ‘units’. The unit was a measure first used in the 1970s to allow for comparison in longitudinal surveys of drinking levels (Ball, Williamson, & Witton, 2007; Herrick, 2011, p. 156–158). A unit of alcohol was equal to 10 ml or 8 g of pure alcohol, or about half a pint of beer.

The HEA’s use of the unit, and the levels at which safe drinking were set, were in line with recommendations made in a series of reports published in 1986–1987 by the Royal College of Psychiatrists, the Royal College of Physicians, and the Royal College of General Practitioners. Each report suggested that sensible limits to drinking equated to 21 units a week for men and 14 units a week for women. The Royal College of Physicians report, *A Great and Growing Evil*, set out a wide range of health and social consequences resulting from the over-consumption of alcohol. The report suggested that the more alcohol consumed, the greater the risk (Royal College of Physicians, 1987). By its very nature, the setting of these limits was, however, somewhat arbitrary. Although the guideline levels were related to the relative risk of cirrhosis of the liver, as many critics have pointed out, the evidence tying this to specific amounts of alcohol was unclear (Ball et al., 2007). As Herrick notes, ‘delineating and communicating the risk thresholds of drinking is far from an exact science’ and these are ‘influenced by range of factors outside public health and biomedicine.’(Herrick, 2011, p. 160) In a much cited statement, a member of one of the expert committees involved in setting the limits said that they had ‘plucked a figure out of the air’, although he later asserted that he stood by the committee’s recommendations (Smith, 2007). Guidelines for health behaviours, whether this be in relation to alcohol consumption, healthy eating, or anything else, present a scientific consensus when this does not necessarily exist. Although such recommendations are based on evidence, this is often contested (Lindsay, 2010). The production of healthy living guidelines is a political process, related to epidemiological evidence of risk, but always open to interpretation and contestation. The sensible drinking limits were intended to provide a guideline that the public could easily understand, and the unit system meant that individuals could be more readily located along a continuum of harmful drinking, something which also allowed the size and scale of the national drinking problem to be assessed (Nicholls, 2009, p. 212–213; Thom, 1999, p. 129–130). Units, and recommended safe drinking levels, despite their flaws, thus served a variety of purposes beyond health education and appeals to individuals to change their behaviour.

Communicating what a unit consisted of, and how this related to risk and safe or sensible levels of drinking, rapidly became a key feature of alcohol health education efforts. This can be observed in the Drinkwise campaign, which was a joint initiative run by the HEA and the charity Alcohol Concern. The campaign was focused around a national Drinkwise day, the first of which took place in June 1989, and the second in June 1990. The Drinkwise day consisted of local and national events such as reports and campaigns, conferences, alcohol-free drink tastings, competitions and quizzes. Local organisers were given packs of materials including posters, leaflets, stickers and balloons to distribute. The aim of the campaign was to ‘offer the general public an opportunity to look at their drinking habits and consider healthy drinking choices.’ The central theme was to ‘improve individual awareness’, get people to become a ‘better judge of your consumption’ and to know ‘your limits for sensible drinking’ (Health Education Authority & Alcohol Concern, 1990). A document providing advice to local organisers on how to run a Drinkwise day stated that ‘Given the social acceptability of drink and low awareness of recommended drinking limits … the “tone of voice” in our communications is extremely important’. The campaign was intended to credit ‘individuals with responsibility and judgement. It is not authoritarian, dictatorial or critical.’ The aim was to increase awareness of sensible drinking levels and prompt ‘objective self-assessment’ (Health Education Authority & Alcohol Concern, 1990).

**Figure 2:**
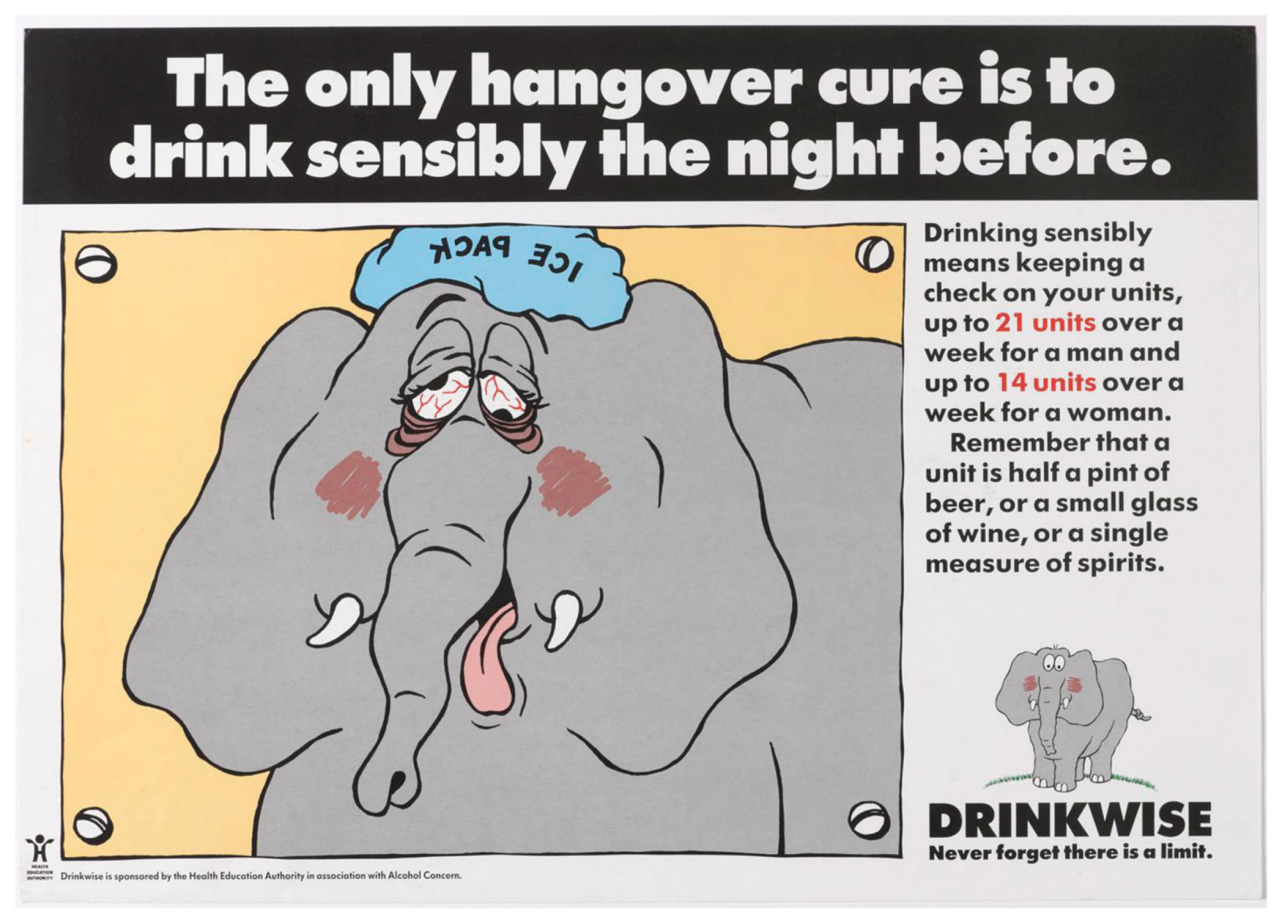
‘The only hangover cure is to drink sensibly the night before’, HEA/Alcohol Concern, 1990. Image courtesy of the Science Museum Group. This image is released under a Creative Commons Attribution-NonCommercial-ShareAlike 4.0 Licence.

**Figure 3:**
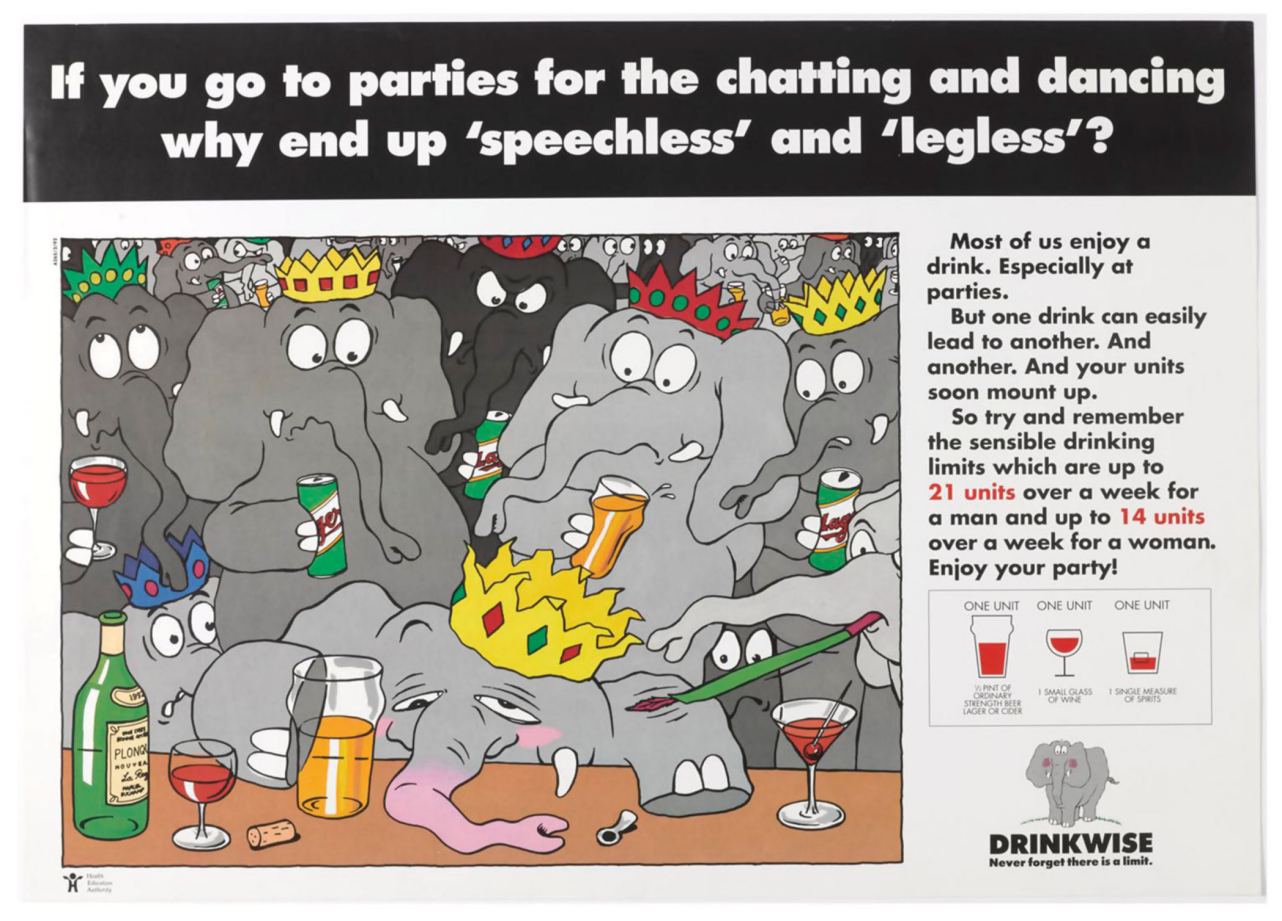
‘If you go to parties for the chatting and dancing why end up “speechless” and “legless”?’ HEA/Alcohol Concern, 1990. Image courtesy of the Science Museum Group. This image is released under a Creative Commons Attribution-NonCommercial-ShareAlike 4.0 Licence.

Elements of this approach can be seen in the campaign materials.^1^ These were anchored around various cartoon drawings of an elephant and the tagline ‘Never forget that there is a limit.’ The image of the elephant, the campaign’s designers asserted, ‘contains the warmth and humour essential to conveying the acceptance of drink in society.’ The precise reasons for the choice of the elephant logo are somewhat opaque, but there are all sorts of associations between elephants and alcohol. Elephants in the wild supposedly get drunk on rotting fruit, although this has more recently been called into question (Morris, Humphreys, & Reynolds, 2006). There is a long running idea that people experiencing drunken hallucinations see pink elephants, something famously played on in the 1941 Disney film, Dumbo (Brown, 2014). The primary reason for choosing the elephant was likely because elephants are supposed to have excellent memories, a motif that reinforced the message that one should ‘never forget there is a limit’. Through the elephant motif, the Drinkwise campaign was encouraging the development of a process of constant self-monitoring to drink sensibly, which was framed as within unit-based guidelines. The consequences of not doing so were represented in various ways, including the immediate results of drinking too much – such as embarrassing behaviour and a hangover (Figure 2). Other images in the campaign played further on forgetting, and on doing things that individuals might wish to forget whilst drunk, something which could be avoided by remembering the sensible drinking limits (Figure 3). Many of the posters also included a reference to the unit-based guidelines and a visual reminder of what a unit consisted of. The aim was to increase awareness of sensible drinking levels and prompt ‘objective self-assessment’. It was not about getting people to drink less per se, but rather to encourage reflection and raise awareness. Indeed, the Drinkwise campaign was set within the context of other activities designed not just to get people to think about how much they drank, but to live healthier lives overall.

**Figure 4:**
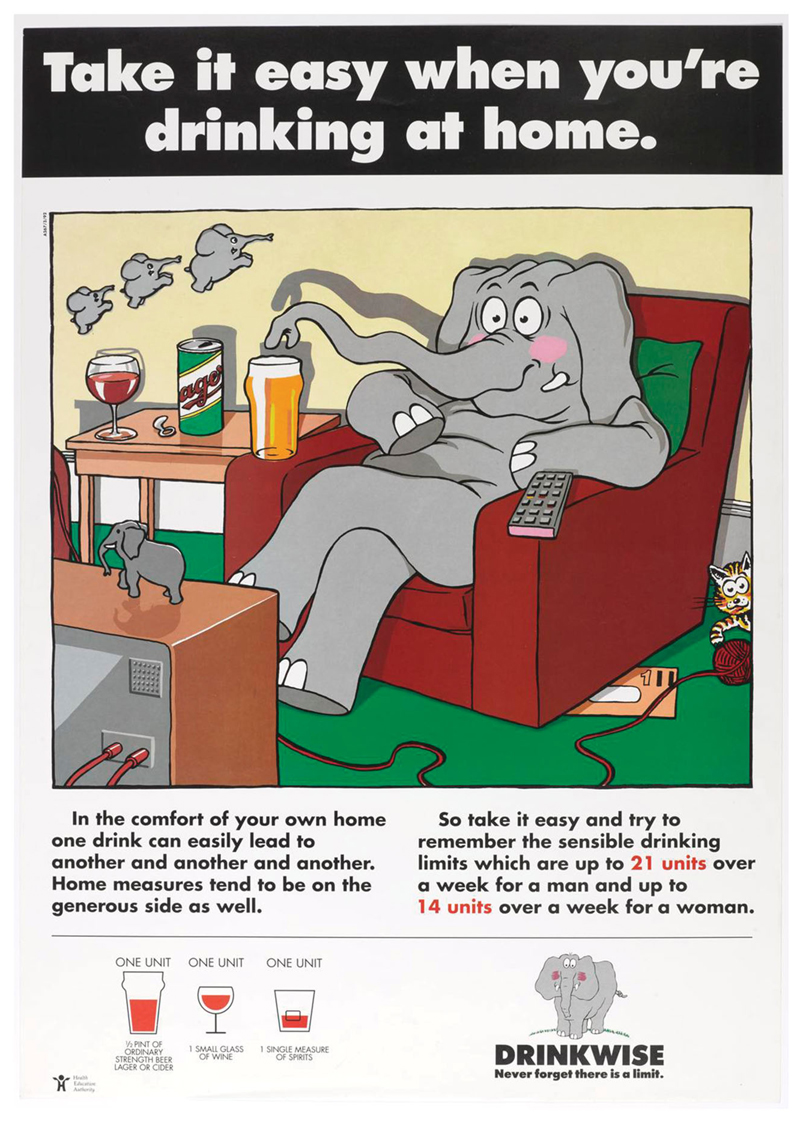
‘Take it easy when you’re drinking at home’, HEA/Alcohol Concern, 1990. Image courtesy of the Science Museum Group. This image is released under a Creative Commons Attribution-NonCommercial-ShareAlike 4.0 Licence.

**Figure 5:**
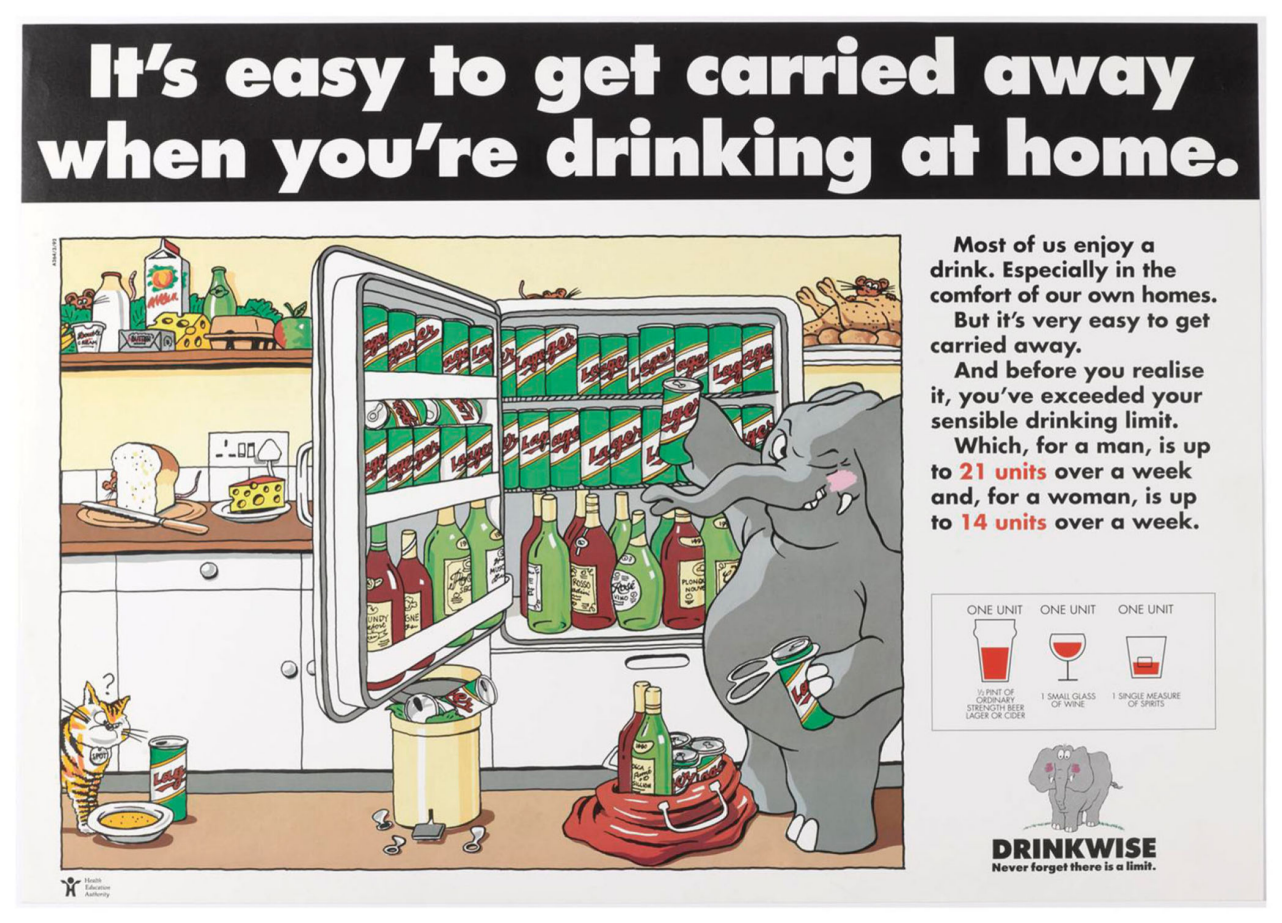
‘It’s easy to get carried away when you’re drinking at home’ HEA/Alcohol Concern, 1990. Image courtesy of the Science Museum Group. This image is released under a Creative Commons Attribution-NonCommercial-ShareAlike 4.0 Licence.

Yet, beneath the softly, softly approach of the Drinkwise campaign, other currents can be detected. Two posters, for instance, targeted drinking in the home (Figures 4 and 5). This was a reflection of changing patterns of alcohol consumption. From the mid-1980s onwards, the amount of alcohol consumed off-license increased as the amount of alcohol consumed in licensed premises decreased (Foster & Ferguson, 2012). The Drinkwise campaign’s posters not only reflected a legitimate concern with home drinking but also an expansion of the sphere of interest from public drinking and drunkenness to more hidden, private consumption. This was representative of a further widening of the targets of alcohol education campaigns. These were no longer just about what was or was not excessive drinking and who might be indulging in it but also the places where excessive drinking might occur. This fitted within a broader direction of travel whereby drinking too much alcohol was seen as a risk for all drinkers and the wider population, not just those who demonstrably had a problem with drinking. All drinkers were potentially at risk, a shift from earlier material produced during the 1970s which focused on alcoholics and heavy drinkers.

Nonetheless, this was often a rather restricted notion of risk. Health education efforts like the Drinkwise campaign had a tendency to focus on the short-term, immediate consequences of drinking too much, such as drunkenness and hangovers, rather than the longer-term health effects. Moreover, the light-hearted tone of some of the materials may have further served to undermine the gravity of the message. The use of humour was a recognition of the social and cultural importance of alcohol consumption within British society, as well as its economic value, and the need to tread carefully to avoid turning people off altogether. But such an approach could also pose difficulties, as it contributed to a sense that the over-consumption of alcohol was not be taken entirely seriously, or at least not as seriously as other health issues. Yet, at the same time, individuals were being called upon to take more responsibility for their drinking and its effects. Health education around alcohol could, therefore, present a conflicting message.

## Limits to limits

By the 1980s, the limitations of health education for dealing with alcohol-related problems were becoming more widely understood. The HEC itself recognised that health education alone would not be able to significantly reduce alcohol consumption or change drinking behaviours. In their alcohol education programme strategy for 1982–1983, the HEC asserted that ‘health education **by itself** [original emphasis] has only a limited ability to reduce [alcohol consumption].’ Instead, the Council suggested that ‘Health education must therefore concentrate on what it does best which almost certainly does not include directly affecting consumption on a mass scale but, rather, working indirectly through creating a climate of opinion’ (HEC, 1982). This crisis of confidence was not just restricted to alcohol health education but was part of a wider questioning of the value of both the HEC and health education more broadly. An editorial in the *British Medical Journal* published in 1982 argued that the HEC had achieved little since it was established in 1968, and that it needed to ‘shift its emphasis from threatening people with the horrors that await them if they continue to smoke and drink to convincing them of the benefits of full health’ (Anon - British Medical Journal Publishing, 1982). Adrian Pollitt, Chief Administrative Officer at the HEC, was well-aware of such criticisms and, at least in part, agreed with them. He noted that in 1979 the HEC had published a monograph titled *Is Health Education Effective?* and ‘In what one might regard as a spectacular own goal, the answer it gave was only a slightly qualified “No”’ (Pollitt, 1984). Health educators themselves were developing a more critical view of their work which stressed the importance of social context and rejected a sole focus on individual behaviour change as a way to improve the public’s health (Rodmell & Watt, 1986).

In the case of alcohol, this led the HEC and others to examine approaches that aimed to address alcohol consumption at the population level rather than on an individual basis. The damage alcohol could cause within a population had been known since at least the 1950s. This was when the French demographer Sully Ledermann contended that the level of alcohol consumption within a population was related to the extent of alcohol problems within that population. As the total amount of alcohol consumed increased, so too did the number of individuals with alcohol problems. The Ledermann thesis was influential on the alcohol ‘policy community’ in the UK, and elsewhere (Thom, 1999). By the late 1970s and into the 1980s, there were a series of reports by medical royal colleges and other expert bodies that accepted the Ledermann thesis and called for population level measures to reduce alcohol consumption (Bruun, 1975; Royal College of General Practitioners, 1987; Royal College of Physicians, 1987; Royal College of Psychiatrists, 1979, 1986). Some of these proposals, such as increasing the price of alcohol (or at least not allowing it to decrease in real terms) were politically controversial. An internal government think-tank report noted that health education would have a limited impact on reducing alcohol consumption, and instead recommended the introduction of control measures such as using tax to increase the price of alcohol and other restrictions on the availability of drink. However, the government was reluctant to use tax policy in this way and were fearful of the economic impact such measures would have on the drinks industry, tax revenue, and jobs. The DHSS wanted to avoid the introduction of what they saw as ‘unacceptably paternalistic policies’ (DHSS, 1983d). Publication of the report was supressed, although it was eventually appeared in Sweden in 1982 (Central Policy Review Staff, 1982). The HEC wanted to quote extracts from the clandestine report in their 1983–1984 alcohol strategy, but the DHSS instructed them not to (DHSS, 1982b; HEC, 1982).

The HEC, however, were not to be deterred. They pursued other tactics to try and push the DHSS to adopt more restrictive policies on alcohol. One of these methods was to support the establishment of a pressure group, Action on Alcohol Abuse (AAA) in 1982. The HEC’s Director General (David Player) was instrumental in creating the AAA, and he was a member of the organisation, something the DHSS were slightly uncomfortable with, but could do nothing about (DHSS, 1983a). Tension flared when the HEC wanted to divert some of their funds towards supporting AAA. The DHSS thought that it was inappropriate for the HEC to finance a campaigning organisation. The HEC pointed out that the government supported Action on Smoking and Tobacco, to lobby against smoking (Berridge, 2007). The DHSS, however, felt that ‘the analogy was a weak one – all smoking was harmful: not all drinking was. A campaigning body would find it difficult to campaign against abuse only as distinct from consumption: a distinction that had to be maintained with alcohol although not with tobacco’ (DHSS, 1983c). Officials told the HEC that ‘Public campaigns to change Government policy are not the business of a Government-funded organisation’ (DHSS, 1983b). Despite this warning, the HEC and the AAA lobbied for greater restrictions or even a ban on the advertising of alcohol. The DHSS countered that ‘Government was most unlikely to be willing to control the advertising of a product which was only harmful if misused.’ Instead, they encouraged the HEC ‘to explore the possibility of co-operation with the industry to produce advertising which reflected socially acceptable values e.g. not to drink and drive or operate machinery’ (DHSS, 1983g).

Indeed, the DHSS wanted the HEC to work much more closely with the alcohol industry. The DHSS thought that the drink manufacturers could be persuaded to help pay for health education materials, although for their part the HEC ‘observed that the sums the industry was spending on preventive measures were small in relation to drinks advertising.’ The HEC ‘agreed that while their general interests and those of the industry were on opposite sides there were common interest areas where co-operation was possible e.g. underage drinking; drinking problems within the trade and drinking and driving’ (DHSS, 1983c). Some of this tension spilled over into the public arena when it was suggested that the Health Minister, Kenneth Clarke, had written to the Chairman of the HEC encouraging the organisation to increase their involvement with ‘industrial interests.’ Clarke asserted that the HEC had not been pressured to accept funds from any interests that would prejudice their work. He said ‘There was no question of the department encouraging the council to accept tobacco sponsorship. But there were other industries which it could approach for funds, such as alcohol, soft drinks and contraceptives’ (Phillips, 1983). The role of the drinks industry in influencing alcohol policy has attracted a good deal of interest in recent years (Hawkins, Holden, & McCambridge, 2012; McCambridge, Mialon, & Hawkins, 2018; Petticrew, Maani Hessari, Knai, & Weiderpass, 2018). At this point, it would appear that there was no clear evidence of direct lobbying by the alcohol industry to prevent the introduction of stronger control measures. Rather, the DHSS were attempting to steer a course between putting measures in place that would help combat alcohol related health problems and at the same time continue to allow the majority of non-problematic drinking to continue. The issue was not so much vested interests as the need to balance imperfectly understood risks with the benefits many associated with ‘sensible’ alcohol consumption.

## Conclusion

The establishment of ‘sensible’ drinking limits and the communication of these to the public during the 1980s was a fraught process. Defining what a safe or sensible amount of alcohol to drink was problematic because of a lack of clear evidence to precisely correlate levels of alcohol consumption with harm. Epidemiological research indicated that as alcohol consumption went up, so too did rates of liver cirrhosis and other alcohol-related conditions, but it was difficult to translate this into guidance for individuals to follow.

But this was more than a problem of lack of evidence. Setting limits was difficult because alcohol consumption was embedded within society and the economy. The Government was reluctant to ‘adopt unacceptably paternalistic policies’ and constrain the legitimate pleasure of many (DHSS, 1983d). This conflicted sense of both the need to encourage individuals to curb their drinking and at the same time recognise the place of alcohol in society carried over into the health education materials designed to foster ‘sensible’ drinking. These often displayed a hazy sense of the risks associated with drinking too much. This was rooted in both the scientific uncertainty about the precise nature of the risks posed, but also the perceived need to tread carefully when communicating with ‘regular’ drinkers. Unlike the material produced during the 1970s targeted at ‘problem’ drinkers, the tactics used, including humour and a light-hearted tone, displayed a recognition that drinking was socially and culturally shaped.

What all of this suggests is that there were clearly ‘limits to limits’ as both a communication tool and as a way to frame and understand the risks associated with alcohol consumption at the individual and population level. Alcohol health education campaigns from the 1980s onwards were largely directed towards individual self-regulation of consumption and how this could be monitored using alcohol units. There were, and continue to be, many problems with the unit system: it is poorly understood by the public and ignored by a significant number of drinkers who regularly exceed the safe drinking limits (Ball et al., 2007; Herrick, 2011). These limits are still somewhat arbitrary: they are not mapped precisely on to relative risk, different countries set different safe drinking levels, and so on. Moreover, placing responsibility on the individual to control their alcohol consumption has a tendency to ignore the structural and environmental influences on drinking and other health behaviours, such as, the role of the alcohol industry. All of this means that it could be suggested that public health campaigns on alcohol were being set up, if not to fail, then at least to only achieve a limited set of objectives. This was something that health educators tacitly recognised, when then they asserted that campaigns were not directed towards reducing drinking levels, but rather changing attitudes towards alcohol consumption. This is not an argument to abandon health education campaigns around alcohol, but rather to think more clearly about the messages being communicated and their potential effects. Close analysis of previous campaigns, and the debates that surrounded these, can tell us much about how future efforts might be improved. In this way, the past can be a guide to the present.

## Footnotes

1
**Note**
It may be possible to dig more deeply into the decisions behind the Drinkwise campaign once the cataloguing of the HEA’s papers at the Wellcome Library is complete.
